# Exploring Molecular Genetic Alterations and *RAF* Fusions in Melanoma: A Belvarafenib Expanded Access Program in Patients with *RAS/RAF*-Mutant Melanoma

**DOI:** 10.1093/oncolo/oyae018

**Published:** 2024-03-12

**Authors:** Kyoo Hyun Kim, Sungmin Cho, Yeyeong Jeong, Eun Sil Baek, Chung Lee, Hyang-Joo Ryu, Young Su Noh, Yoon-hee Hong, Kee Yang Chung, Mi Ryung Roh, Byung Ho Oh, Chang Gon Kim, Minkyu Jung, Sang Joon Shin

**Affiliations:** Division of Medical Oncology, Department of Internal Medicine, Yonsei Cancer Center, Yonsei University College of Medicine, Seoul, Republic of Korea; Department of Medicine, Yonsei University College of Medicine, Seoul, Republic of Korea; Department of Medicine, Yonsei University College of Medicine, Seoul, Republic of Korea; Songdang Institute for Cancer Research, Yonsei University College of Medicine, Seoul, Republic of Korea; Department of Pathology, Yonsei University College of Medicine, Seoul, Republic of Korea; Department of Pathology, Yonsei University College of Medicine, Seoul, Republic of Korea; Clinical Science, Deparment of Clinical Research and Development, Hanmi Pharm. Co., Ltd., Seoul, Republic of Korea; Clinical Science, Deparment of Clinical Research and Development, Hanmi Pharm. Co., Ltd., Seoul, Republic of Korea; Department of Clinical Research Design & Evaluation, SAIHST, Sungkyunkwan University, Seoul, Republic of Korea; Department of Dermatology, Severance Hospital, Yonsei University College of Medicine, Seoul, Republic of Korea; Department of Dermatology, Gangnam Severance Hospital, Yonsei University College of Medicine, Seoul, Republic of Korea; Department of Dermatology, Severance Hospital, Yonsei University College of Medicine, Seoul, Republic of Korea; Division of Medical Oncology, Department of Internal Medicine, Yonsei Cancer Center, Yonsei University College of Medicine, Seoul, Republic of Korea; Division of Medical Oncology, Department of Internal Medicine, Yonsei Cancer Center, Yonsei University College of Medicine, Seoul, Republic of Korea; Division of Medical Oncology, Department of Internal Medicine, Yonsei Cancer Center, Yonsei University College of Medicine, Seoul, Republic of Korea; Department of Medicine, Yonsei University College of Medicine, Seoul, Republic of Korea; Songdang Institute for Cancer Research, Yonsei University College of Medicine, Seoul, Republic of Korea

**Keywords:** high-throughput nucleotide sequencing, melanoma, belvarafenib, RAF fusion

## Abstract

**Background:**

Melanoma incidence is on the rise in East Asia, yet studies of the molecular landscape are lacking in this population. We examined patients with melanoma who underwent next-generation sequencing (NGS) at a single tertiary center in South Korea, focusing on patients harboring *NRAS* or *RAF* alterations who received belvarafenib, a pan-RAF dimer inhibitor, through the Expanded Access Program (EAP).

**Patients and Methods:**

Data were collected from 192 patients with melanoma who underwent NGS between November 2017 and May 2023. Variant call format data were obtained and annotated. Patients in the EAP received 450 mg twice daily doses of belvarafenib.

**Results:**

Alterations in the RAS/RTK pathway were the most prevalent, with *BRAF* and *NRAS* alteration rates of 22.4% and 17.7%, respectively. NGS enabled additional detection of fusion mutations, including 6 *BRAF* and 1 *RAF1* fusion. Sixteen patients with *NRAS* or *RAF* alterations received belvarafenib through the EAP, and disease control was observed in 50%, with 2 patients demonstrating remarkable responses.

**Conclusions:**

Our study highlights the value of NGS in detecting *BRAF*, *NRAS* mutations and *RAF* fusions, expanding possibilities for targeted therapies in malignant melanoma. Belvarafenib showed clinical benefit in patients harboring these alterations. Ongoing trials will provide further insights into the safety and efficacy of belvarafenib.

## Teaser

This article provides an overview of the molecular landscape of malignant melanoma in patients who underwent next-generation sequencing at a single tertiary institution in South Korea, and presents results from a belvarafenib Expanded Access Program (EAP).

**Implications for Practice**

Amidst the rising incidence of malignant melanoma in East Asia, this study provides an insightful overview of the molecular landscape of patients with malignant melanoma who underwent next-generation sequencing at a single tertiary institution in South Korea. We identify potential candidates that are not considered typical candidates for BRAF-targeted therapy, with a focus on patients harboring *BRAF* mutations/fusions or *NRAS* mutations. We highlight the therapeutic benefits of belvarafenib in the Expanded Access Program, particularly for patients with *BRAF* and *NRAS* alterations, an underexplored territory in targeted therapy.

## Introduction

The incidence of newly diagnosed patients with melanoma has risen each year, with 324,635 new cases and 57,043 new deaths in 2020.^1^ Melanoma incidence is also on the rise in East Asia, yet a comprehensive understanding of the molecular landscape is lacking in this population.^2^ Over the past decade, systemic therapies for malignant melanoma have evolved, with standard options including immune checkpoint inhibitors and molecularly targeted therapies for advanced-stage disease with regional or distant metastases.^3^ Notably, patients harboring *BRAF* V600 activating mutations are now endowed with expanded treatment options of BRAF and MEK kinase inhibitor combinations. Based on results from phase III trials, currently approved drug combinations include vemurafenib with cobimetinib, dabrafenib with trametinib, and encorafenib with binimetinib.^3-7^ Also, given the higher prevalence of c-*KIT* alterations observed in the Asian populations, KIT inhibitors such as imatinib, nilotinib, and regorafenib may be considered for patients harboring c-*KIT* mutations.^8-12^ However, for individuals with other non-*BRAF* mutation subtypes or *NRAS* mutations, targeted therapies are currently lacking, leaving most of these patients reliant on immune checkpoint inhibitors and cytotoxic chemotherapy options.

According to The Cancer Genome Atlas (TCGA) data, melanoma can be genomically subdivided into the following 4 groups based on the mutated genes; mutant *BRAF,* mutant *RAS,* mutant *NF1*, and triple-wild type (Triple-WT) groups.^13^ Conventionally, *BRAF* mutation was assessed via polymerase chain reaction (PCR)—based assay, but with the advent of expert gene panels, next-generation sequencing (NGS) has emerged as a more accessible and high-throughput method. NGS has become a cornerstone of precision medicine, prompting clinicians to use it early in cancer diagnosis and treatment on a patient-specific basis.^14,15^ Compared to the conventional, single-gene targeted, PCR-based methods, NGS, which incorporates targeted RNA sequencing, also has the advantage of identifying fusion genes, particularly those with novel fusion partners not targeted by probes.^16^

Belvarafenib (HM95573) is a pan-RAF dimer inhibitor known to inhibit both BRAF and CRAF monomers/homodimers/heterodimers.^17,18^ In phase Ib trial of belvarafenib in combination with cobimetinib for patients with advanced solid tumors harboring either *NRAS* Q61 or *BRAF* V600 mutations, promising results in terms of efficacy and tolerability were reported, including those who had previously received BRAF and MEK inhibitor combinations.^19^ Furthermore, recent studies have suggested that belvarafenib may have the potential for treating patients with brain metastases, as it has shown high brain/plasma concentration in preclinical models.^20^ In line with these preclinical and clinical trial results, patients with *NRAS, RAF* mutations or fusions without currently available therapeutic options have been granted access to belvarafenib through the Hanmi Expanded Access Program (EAP) program in South Korea.

In this study, we conducted a comprehensive analysis of patients with malignant melanoma who had undergone NGS testing at our institution, further highlighting clinical experiences from the subset of patients who, through either PCR or NGS, were found to harbor potentially targetable mutations and received belvarafenib monotherapy through the EAP program. Here, we report real-world experiences and share perspectives on the potential of *RAF* fusions and *NRAS* mutations as clinically actionable genomic alterations.

## Methods

### Patient Data Collection

Data for patients with advanced malignant melanoma, diagnosed and followed up at a single academic institution (Yonsei Cancer Center, Seoul, South Korea) between November 2017 and May 2023, and whose next-generation sequencing (NGS) data were available, were obtained.

Patient demographics and clinical information were obtained from the clinical database maintained by the YCDL (Yonsei Cancer Data Library) through the medical records at the Yonsei Cancer Center, Severance Hospital. Patient characteristics including age, sex, primary tumor subtype, sites of metastases, tumor stage based on the 8th edition staging system of the American Joint Committee on Cancer (AJCC), and previous treatment including surgery, radiotherapy, and systemic treatment were collected. Results of routine molecular testing other than NGS, including *BRAF* and *NRAS* polymerase chain reaction (PCR) assays, were also noted. If available, clinical survival data including the date of disease progression after each line of therapy and the date of death were also collected.

### Molecular Analysis

#### Next Generation Sequencing

Since November 2017, patients with advanced-stage malignant melanoma have been offered the option of NGS testing for targetable molecular alterations. Briefly, patient tumor cells were isolated from formalin-fixed and paraffin-embedded (FFPE) specimens and DNA was extracted using standard techniques. NGS was performed using a hybridization-based capture platform (TruSight Oncology 500 Kit; Illumina). Patient NGS data were obtained in Variant Call Format (VCF) files which served as the primary input data for further analysis.

#### Mutation Calling (Variant Annotation)

The sequenced reads were aligned to the human genome assembly (hg19) using Pisces (v.5.2.11.63). The initial aligned VCF files underwent further quality score-based filtration using SAMtools and BCF tools, applying specific criteria, including minimum read quality (read depth) and minimum variant frequency. Variants were filtered to retain those meeting stringent quality thresholds of variant quality > 20 and variant allele frequencies (VAF) > 0.1%.

To enhance the robustness of variant annotations, we used AnnoVar.^21^ gnomAD (version 2.1.1, available at https://gnomad.broadinstitute.org/),^22^ ClinVar (clinvar_20220320/hg19, accessible at https://www.ncbi.nlm.nih.gov/clinvar/),^23^ COSMIC (cosmic v70, https://cancer.sanger.ac.uk/cosmic),^24^ and dbSNP (dbSNP Build138, https://www.ncbi.nlm.nih.gov/snp/)^25^ data sets were used as a reference database for known polymorphic sites and clinical significance. To ensure the exclusive inclusion of clinically relevant variants, we selectively extracted variants annotated as “Pathogenic,” “Likely pathogenic,” “Conflicting interpretations of pathogenicity,” “Drug response” from the AnnoVar files.

R-package “maftools” (version 2.14.0) was used to merge each annotated VCF file into a Mutated Annotation Format (MAF) file and for subsequent visualization and comprehensive summarization.^26^

#### Pathway Map

The visualization of the enrichment pathway was carried out using pathway mapper, a web-based visualizing tool that encompasses a range of cancer-related pathways.^27^ Each pathway data was based on TCGA Pan-Cancer pathways, and the alteration frequency data was extracted from the original MAF file.

### Belvarafenib Treatment Through the EAP

Through an EAP, patients harboring *RAF* or *RAS* mutations as determined by either NGS or PCR assay, and who had experienced standard therapy failure, were granted access to single-agent belvarafenib. Patients were not required to have a measurable target lesion at the start of belvarafenib therapy.

The treatment regimen consisted of oral administration of 450 mg of belvarafenib twice daily, administered in 28-day cycles. Patients underwent regular imaging assessments, such as computed tomography (CT) or magnetic response imaging (MRI), at 8- to 12-week intervals to monitor treatment response. Response analysis was based on Response Evaluation Criteria in Solid Tumors (RECIST) version 1.1. Treatment continued until disease progression, death, or at the patient’s request to discontinue, whichever came first.

### Statistical Analysis

Descriptive statistics were used to summarize patient demographics. Categorical and continuous variables were compared using the chi-square test and unpaired *t* tests, respectively. Progression-free survival (PFS) denoted the time from treatment initiation to disease progression or death, while overall survival (OS) measured the time from treatment initiation to all-cause mortality. Data were censored at the last observation point in case of tracking discontinuation before disease progression or death, treatment cessation due to toxicities, regimen changes, or withdrawal of consent.

## Results

### Baseline Characteristics

From November 2017 to May 2023, data were acquired from 192 patients with advanced-stage malignant melanoma who underwent NGS testing for targetable molecular alterations at Yonsei Cancer Center (YCC). Patient demographics and baseline clinical characteristics are described in Table 1. The median age at diagnosis was 60 years, with a slightly higher number of female (53.6%) patients. Patient with melanoma subtypes included acral melanoma in 62 (32.3%), mucosal melanoma in 61 (31.8%), and cutaneous melanoma, including both chronic sun damage (CSD) and non-CSD melanomas in 39 (20.3%) within the study population. Patients with choroidal melanoma and melanoma with unknown primary origin were also included.

**Table 1.** Baseline characteristics.

Characteristics		No. of patients *N* = 192	%
Median age at diagnosis (IQR)		60	(50-67)
Age at NGS (IQR)		63	(52-69)
Gender	Male	89	46.4
	Female	103	53.6
Subtype	Acral	62	32.3
	Mucosal	61	31.8
	Cutaneous^a^	39	20.3
	Ocular	11	5.7
	Unknown primary	19	9.9
Primary tumor site	Skin	99	51.6
	Mucosa	58	30.2
	Eye	13	6.8
	Others^b^	7	3.6
	Primary site unknown	15	7.8
Disease stage, at diagnosis	CIS	3	1.6
	I	16	8.3
	II	57	29.7
	III	51	26.6
	IV	30	15.6
	Unknown	35	18.2
			
Prior radiotherapy	Yes	157	81.8
	No	35	18.2
			
Prior surgery	Yes	171	89.1
	No	21	10.9
			
Timing of NGS	At baseline	46	24.0
(No. of prior systemic therapy)	1	67	34.9
	2	42	21.9
	3	25	13.0
	4	11	5.7
	5	1	0.5

^a^Cutaneous subtype includes both chronic sun damage (CSD) and non-CSD subtypes.

^b^Others include melanoma primarily in the lung, esophagus, neck soft tissue, urethra, abdomen, and bone.

At the time of molecular profiling via NGS, 76.0% (146/192) of the patients had received at least one line of systemic therapy, and 41.1% (79/192) had received 2 or more lines of therapy.

### NGS Detection of Potentially Targetable Genetic Alterations: A Comparative Analysis With Conventional PCR

**Figure 1.**
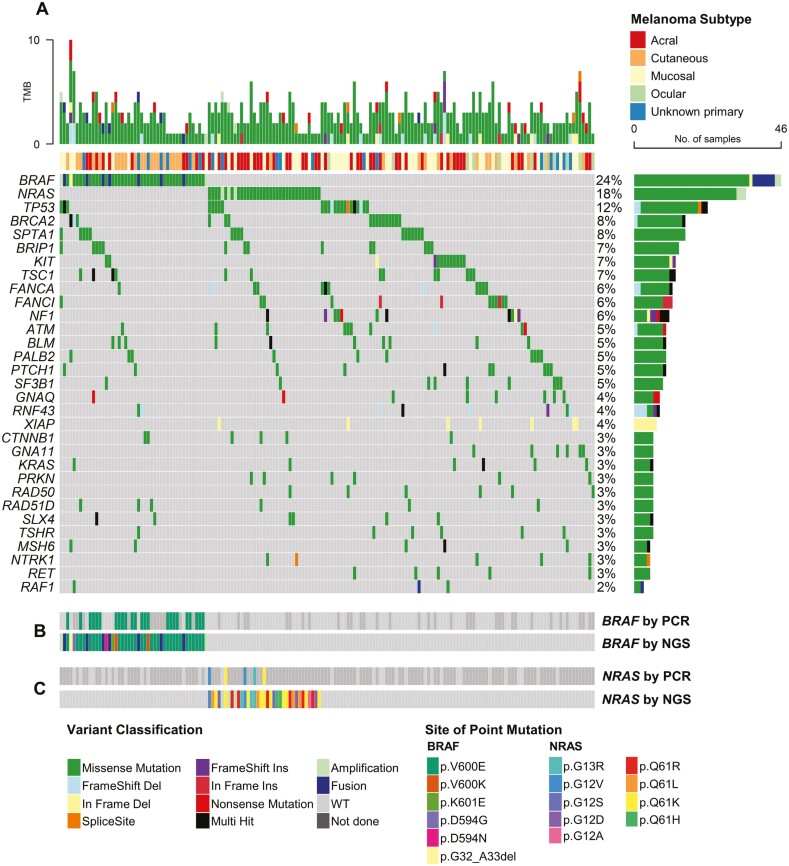
Mutational landscape of melanoma. (**A**) Tumor mutational burden (top), melanoma subtype of each patient (middle), and oncoplot displaying individual mutations and copy number alterations (bottom). (**B**) *BRAF* mutations, as detected either by polymerase chain reaction (PCR)-based Sanger sequencing (top) or next-generation sequencing (NGS) (bottom). (**C**) *NRAS* mutations, as detected either by PCR Sanger sequencing (top), or NGS (bottom).

The spectrum of driver gene alterations, as determined by NGS, is visually represented in Fig. 1A. Special consideration was given to well-known and prevalent mutations such as *BRAF* and *NRAS*, which are potential targets for belvarafenib. We compared the mutation rates of *BRAF* and *NRAS* detected using the PCR-based and NGS assays (Fig. 1B and 1C; Supplementary Fig. S1).

Out of the 192 patients, 145 had undergone conventional, PCR-based *BRAF* mutation testing, with the majority being screened before NGS. The remaining 47 patients had opted for upfront NGS testing without a *BRAF*–PCR test. The prevalence of *BRAF* mutation by PCR was 16.6% (24/145). Overall, NGS detected *BRAF* gene aberrations in 24% of the total patients (46/192), including non-V600 mutations, fusions, and amplifications in 2 (1.0%), 6 (3.1%), and 2 (1.0%) patients, respectively. Interestingly, two patients initially classified as wild type by PCR were later revealed to have *BRAF* alterations by NGS, including one patient harboring *BRAF* amplification.

The detection of *NRAS* mutations using PCR was less frequent, performed in only 25% of the total patients (48/192), with a mutation incidence of 10.4% (5/48). By NGS, we detected *NRAS* mutations in 34 cases (17.7%), including G12/G13 point mutations, Q61 point mutations, and amplifications in 5.2%, 10.9%, and 1.6%, respectively. All *NRAS* mutations detected by PCR were also identified by NGS.

We noted the fraction of patient cases with at least 1 alteration in each of the following signaling pathways: RTK/RAS, cell cycle, PI3K, TP53, Notch, Wnt, Myc, Hippo, TGFbeta, and Nrf2. The most frequently altered oncogenic signaling pathway was the RTK–RAS signaling pathway (*n* = 100, 51.8%) which exhibited the highest median frequency of alterations among our cohort of 192 patients. Other commonly altered pathways included the TP53 pathway (8.3%), cell cycle pathway (7.3%), and PI3K pathway (7.8%). Altered pathways are depicted in Supplementary Fig. S2.

### Patients With *RAS* and *RAF* Fusion—Response to Pan-RAF Inhibitor Belvarafenib

Through an expanded access program (EAP), patients with either *RAF* or *RAS* alterations were granted access to belvarafenib monotherapy after individual case evaluations. Since the first patient in January 2021, the EAP program has provided belvarafenib for 16 melanoma patients harboring various *RAS* and *RAF* mutations as shown in Table 2, including 11 patients with *NRAS* missense mutations, 2 patients harboring *BRAF* missense mutations, and 3 patients with *RAF* fusions (*LMBR1*–*BRAF*, *AGK*–*BRAF*, *MIPOL*–*RAF1*). All participants had undergone at least 1 prior line of palliative systemic treatment, except for 1 patient who experienced disease progression after adjuvant pembrolizumab and was subsequently treated with belvarafenib as first-line therapy. The median number of prior lines of systemic therapy was 2.5. The majority of the patients (14/16, 87.5%) had previously been exposed to immunotherapy.

**Table 2.** Belvarafenib response in patients with malignant melanoma harboring RAS or RAF mutations.

No.	Sex	Age	Primary site	Subtype	Target mutation	Prior lines of therapy	Months of treatment	Best overall response	Time to progression [months]	Survival [months]	Ongoing status	Survival status
1	F	68	Calf	Cutaneous	BRAF V600E	4	1.5	PD	1.5	3.5	–	Dead
2	F	70	Vulva	Cutaneous	LMBR1-BRAF	3	0.2	NA	0.2	0.2	–	Dead
3	M	16	Unknown (Parotid LN)	Unknown	AGK-BRAF	3	16.6	PR*	16.5	27.9	–	Alive
4	F	66	Sole	Acral	NRAS G13R	4	4.6	SD	4.7	5.3	–	Dead
5	M	66	Sole	Acral	NRAS G12S	3	1.6	NA	–	1.6	–	Alive
6	F	68	Posterior Ethmoid	Unknown	NRAS G12A	2	4.5	PR	4.2	17.4	–	Dead
7	M	50	Heel	Acral	NRAS G61K	4	2.4	PD	1.6	2.3	–	Alive
8	F	45	Unknown (Inguinal LN)	Unknown	NRAS Q61H	2	1.4	PD	1.1	11.4	–	Alive
9	F	51	Skin,Vulva	Mucosal	NRAS Q61L	5	6.6	PR*	6.2	7.8	–	Alive
10	F	51	Nasal cavity	Unknown	NRAS Q61K	2	1.4	PD	1.4	1.5	–	Alive
11	M	75	Heel	Cutaneous	NRAS Q61R	2	1.8	PD	1.5	5.7	–	Dead
12	M	62	Nasal cavity	Mucosal	BRAF D594G, RAF1	2	8.5	PR*	–	8.5	Ongoing	Alive
13	F	67	Skin, Abdomen	Unknown	MIPOL-RAF1	1	5.6	PR	–	5.6	Ongoing	Alive
14	M	65	Heel	Unknown	NRAS Q61R	3	0.3	NA	0.2	0.4	–	Alive
15	M	69	Heel	Acral	NRAS Q61K	0	4.6	SD	–	4.6	Ongoing	Alive
16	M	34	Unknown (brain)	Unknown	NRAS Q61R	1	3.7	SD	–	3.7	Ongoing	Alive

Confirmed responses are marked with an asterisk (*).

Abbreviations: NA, not available; PD, progressive disease; PR, partial response; SD, stable disease.

**Figure 2.**
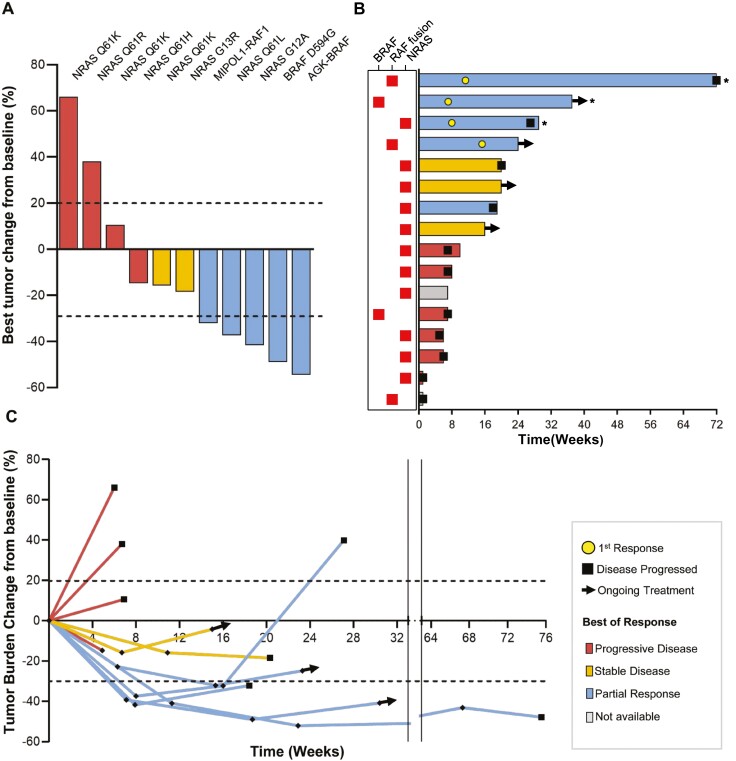
Belvarafenib response in patients harboring *RAS* or *RAF* alterations. (**A**) Waterfall plot showing tumor shrinkage from baseline. *BRAF*, *RAF1*, or *NRAS* alterations are specified above each bar. (**B**) Swimmer plot showing the duration of belvarafenib treatment and responses. Alterations categorized into *BRAF* mutations, fusion involving *RAF*, or *NRAS* mutations are shown in the columns on the left. Confirmed partial response is marked with an asterisk (*). Ongoing patients are marked with arrows. (**C**) Spider plot showing changes in tumor burden from the baseline. Ongoing patients are marked with arrows.

At the time of data cutoff (October 26, 2023), 8 out of the 16 patients (50%) showed either partial response (PR) or stable disease (SD) and confirmed partial responses were noted in 3 patients, with 2 of these patients achieving durable responses lasting longer than 6 months (Fig. 2). Patients 2, 5, and 14, as indicated in Table 2, stopped taking belvarafenib before their response evaluation. These patients had poor performance status at the beginning of belvarafenib treatment, as determined by an Eastern Cooperative Oncology Group (ECOG) score of 2. These individuals had undergone a minimum of 3 prior therapy regimens. Apart from belvarafenib, the discontinuance occurred due to intractable acute renal failure, deterioration in mental status caused by multiple brain hemorrhagic metastases, and deteriorating concomitant renal insufficiency, respectively.

The safety profile of belvarafenib was consistent with the known safety profile of the drug. The most commonly experienced grade III adverse event (AE) was skin rash (4 cases total, 3 patients ≥ G3). Aspartate aminotransferase (AST)/alanine aminotransferase (ALT) elevation and creatinine elevation were commonly reported as well, but all reported AEs were of grades I and II.

Here, we highlight the 2 patient cases with *BRAF* or *RAF1* fusions who had no remaining therapeutic options that have shown remarkable responses to belvarafenib.

#### Case 1

**Figure 3.**
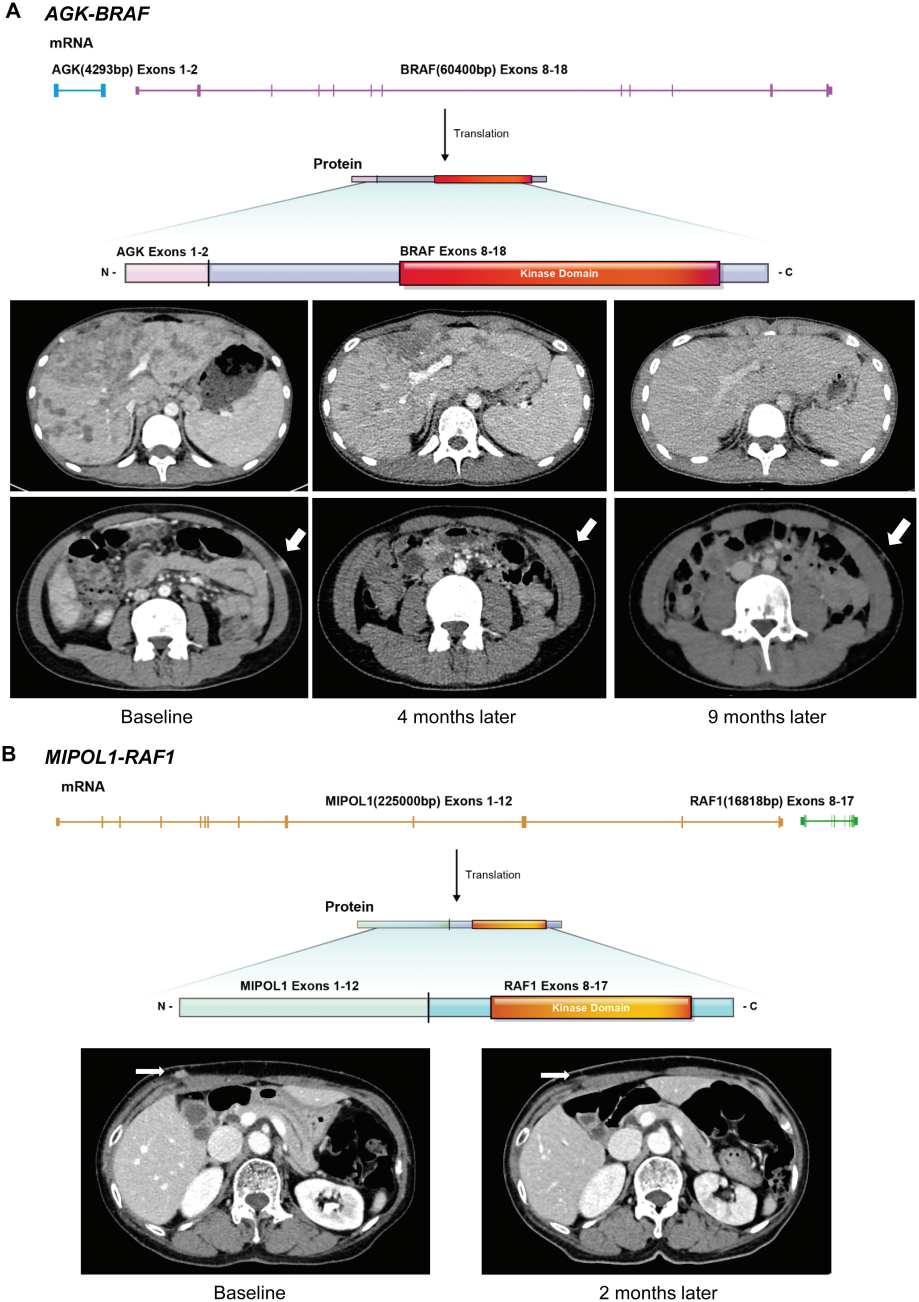
Identification of *RAF* fusions and belvarafenib response. (**A**) Structural details of the *AGK*–*BRAF* fusion identified by NGS (above), and serial computed tomography (CT) images of an patient with *AGK*–*BRAF* fusion who has received belvarafenib treatment, at baseline, 4 months, and 9 months from the start of therapy (below). The upper row indicates diffuse liver metastases in both hemilivers. The bottom row shows a cutaneous lesion on the left flank. The lesion is indicated by an arrow. (**B**) Structural details of *MIPOL1*–*RAF1* fusion (above) and belvarafenib response in a patient with *MIPOL1-RAF1* fusion-positive melanoma (below). The abdominal wall nodule is indicated by an arrow.

The first case was a 15-year-old boy with malignant melanoma with *BRAF* fusion mutation. The patient initially underwent left partial superficial parotidectomy due to the swelling of the parotid glands, and later through additional open biopsy, was ultimately diagnosed with primary unknown malignant melanoma spanning the left parotid gland as well as nearby neck lymph nodes. The patient received proton therapy and 3 lines of systemic therapy including pembrolizumab, dacarbazine, and paclitaxel/carboplatin until imaging follow-up revealed newly developed peritoneal carcinomatosis, liver metastasis, and increased subcarinal lymph nodes. Tissue-based NGS revealed *AGK*–*BRAF* fusion, with the preserved intact *BRAF* kinase domain encoded by exons 11-18 (Fig. 3A), and also *FLT3* (*p.T820N*) and *PALB2* (p.Q987*) mutations. The tumor mutation burden (TMB) was 2.4 per megabase.

Having progressed on all possible systemic treatment options and considering that he was 15 years old at the time, making him ineligible for the majority of clinical trials ongoing at that time, the patient was granted belvarafenib through the EAP program. Upon the start of belvarafenib, the patient showed a dramatic reduction in tumor size, with a notable decrease of –54.5%, and a significant decrease in the size of numerous metastatic lesions in both hemilivers (Fig. 3A). The patient was generally tolerable to the drug, experiencing only mild elevation in AST/ALT (grade I) and creatinine (grade I). The patient remained on belvarafenib and was progression-free for 16.5 months until progression in the pelvic soft-tissue lesions.

#### Case 2

The second case is a 67-year-old female with cutaneous melanoma initially localized in the abdomen. After undergoing an initial wide excision procedure, the disease recurred 7 months later, manifesting as multiple soft-tissue nodules in the anterior abdomen. The patient received first-line pembrolizumab with concurrent radiotherapy; however, disease progression was observed after only 3 treatment cycles. NGS analysis revealed the presence of a *MIPOL1*–*RAF1* fusion, in concurrence with *PTEN* loss and *CDKN2A/B* loss. The kinase-coding domain of the *RAF1* portion of the fusion gene remained intact (Fig. 3B).

The patient was subsequently enrolled in the EAP program and initiated on belvarafenib in April 2023. The patient experienced mild AST/ALT elevation (grade I) and skin rash (grade II), but generally adverse events were tolerable and manageable with hepatotonic agents and topical solutions. The patient demonstrated a partial response (PR), marked by a 32.1% reduction in the size of the abdomen wall lesions (Fig. 3B). The patient remains under ongoing treatment with belvarafenib, as of October 2023.

## Discussion

Comprehensive genomic profiling through NGS broadens the scope of targeted therapeutics in melanoma, by identifying patients with mutations beyond the common BRAF V600E, such as gene fusions. These alterations often elude detection with traditional PCR-based assays, which, consequently, may lead to their exclusion from potential therapeutic options. We have shed light on this aspect in our retrospective analysis of the mutational landscape via NGS in melanoma, particularly focusing on the *RAF* and *NRAS* mutations.

*BRAF* mutations can be typically classified according to the mutation's kinase activity, RAS dependence, and dimerization status; class I mutations involve the V600 codon, class II are non-V600 mutations that are RAS-independent dimers with increased, yet somewhat weaker kinase activity, and class III mutations have low-level or impaired kinase activity, but facilitates RAS binding and CRAF activation.^28^ These non-V600E *BRAF* mutations have been previously reported to be prevalent in 5% to as high as 35% of solid cancer, yet current standard targeted therapeutic options are not available for these mutations.^18^*NRAS* mutations are also prevalent in melanoma but often remain underexplored, with 1 previous phase III trial comparing binimetinib to dacarbazine in *NRAS*-mutant melanoma and demonstrating a modest increase in PFS for binimetinib (2.8 months vs 1.5 months).^29^ These mutations are associated with poor prognosis yet without approved targeted therapies; MEK inhibitors have demonstrated limited clinical benefit, but resistance mechanisms involving the RTK pathway upregulation remain a challenge.^30^

In our retrospective cohort of patients with melanoma subjected to NGS, we observed not only a strong correlation between NGS and PCR-based methods for detecting *BRAF* and *NRAS* mutations but also revealed instances of falsely identified wild-type cases by PCR, including detection of copy-number gain or amplification mutations. The prevalence of *BRAF* and *NRAS* mutation rates in our study—22.4% and 17.7%, respectively—was higher than the rates of 17.6% and 12.6% reported in a prior study involving a Korean patient with melanoma population utilizing PCR-based assays.^31^ While mutation prevalence may vary from one study to another and drawing concrete conclusions may be difficult, it is plausible to suggest that the increased prevalence of *NRAS* and *BRAF* mutation in our cohort may be attributed to the heightened sensitivity and accuracy of the NGS.

In addition to the *BRAF* and *NRAS* mutations, our NGS analysis revealed a total of 8 patients harboring either *BRAF* or *RAF1* fusion mutations, each with distinct fusion partners (*AGK–BRAF, CUX1–BRAF, TRIM24–BRAF, BRAF–DPP6, GOLG4–BRAF, LMBR1–BRAF, KIAA1548–BRAF, MIPOL1–RAF1*). Notably, 1 patient exhibited concurrent *BRAF–DPP6* and *ESYT2–BRAF* fusions.

*BRAF* fusions, similar to the class II *BRAF* mutation group, induce BRAF dimerization and constitutive activation of the MAPK pathway.^18,32^ Various BRAF fusion partners have been identified in various solid cancers including melanoma, such as *AGK–BRAF, KIAA1549–BRAF, AKAP9–BRAF*, and *TRIM24–BRAF* fusions.^33^ In melanoma, these fusions are more prevalent in females and can arise anywhere on the skin and mucosa.^32,34^ Recent findings suggest that the emergence of *BRAF* fusions may play a role in resistance mechanisms across various solid cancers, including EGFR-mutant lung cancers treated with tyrosine kinase inhibitors, gastric cancer treated with FGFR inhibitors, and *BRAF* V600E-mutant melanomas treated with vemurafenib.^33,35,36^

*RAF1*, also known as *CRAF*, belongs to the same family as *BRAF* and participates in the MAPK signaling pathway and is associated with various cancers including melanoma. Specific mutations, like the *CRAF* R391W mutation, act as driver oncogenes, promoting continuous homodimerization of the protein and increased MAPK pathway activity.^37^ Structure variants involving the *RAF1* gene fusions and their response to MEK inhibition or RAF inhibition through trametinib have been previously reported.^38,39^

With the implementation of NGS to guide treatment decisions, we were able to identify patients with *BRAF* mutations, *RAF* fusions, and *NRAS* mutations who might potentially benefit from belvarafenib. Although our experience with belvarafenib in these patients remains limited and preliminary, we have observed encouraging results, with 50% of the patients achieving disease control. Notably, the case featuring the *AGK–BRAF* fusion stands out, with an exceptional tumor response to belvarafenib in a patient who might otherwise be ineligible for such clinical trials due to their young age. Also, given that the majority of patients in our study had prior exposure to immunotherapy, it appears that previous immunotherapy exposure does not act as a deterring factor in efficacy, but further research is needed to provide a more robust confirmation of this observation.

Expanding our scope to include other solid cancers, based on a case report of urothelial carcinoma featuring a *NRF1–BRAF* fusion that demonstrated a clinical response to trametinib,^39^ we have enrolled a patient with renal pelvis cancer harboring *NRF1–BRAF* fusion into the belvarafenib EAP program. Remarkably, this patient has maintained stable disease while receiving belvarafenib for over 5 months. This observation emphasizes that response to belvarafenib extends beyond patients with melanoma, suggesting its potential efficacy in a broader range of patient populations.

Currently, there are 3 ongoing phase Ib/II trials involving belvarafenib. One phase Ib, the dose-escalation study aims to evaluate belvarafenib in combination with either cobimetinib or cetuximab in patients with locally advanced solid cancer harboring *RAS* or *RAF* mutations (NCT03284502). Another global, phase Ib trial in previous anti-PD1 or anti-PD-L1 treated, patients with NRAS-mutant advanced melanoma aims to compare belvarafenib as a single agent and in combination with either cobimetinib or cobimetinib plus nivolumab (NCT04835805). Lastly, belvarafenib in patients with *BRAF* class II or fusion-positive tumors is studied as part of a larger phase II, platform study (NCT04589845).

Given that the findings reported here are derived from an EAP program designed for compassionate drug use, there are some limitations. Compared with standard clinical trials, EAPs may involve fewer comprehensive data collection and reporting, and long-term follow-up data for these patients is limited, as some patients are currently still receiving treatment. However, while little is known about the long-term response and potential resistance mechanisms to belvarafenib in patients with *BRAF* fusions and other *RAF* mutations, the preliminary findings from the EAP program are promising. Furthermore, NGS in the current and upcoming decade will continue to remain a pivotal tool in identifying potential responders to targeted therapies like belvarafenib. Based on our experiences, we believe that continued clinical research with belvarafenib will significantly contribute to the advancement of precision medicine in melanoma treatment.

## Conclusion

Our retrospective study has highlighted the value of NGS in detecting *BRAF, NRAS* mutations and *RAF* fusions, extending the possibilities for targeted therapies in malignant melanoma. Belvarafenib is a promising, potential treatment option for patients with these genetic alterations. Ongoing trials will provide additional insights into the efficacy and safety of belvarafenib and its role in precision medicine-based treatment strategies for patients with melanoma.

## Supplementary Material

oyae018_suppl_Supplementary_Figures

## Data Availability

The datasets used and/or analyzed during the study are available from the corresponding author on reasonable request.
