# Supplementary material for: Exploring Molecular Genetic Alterations and RAF Fusions in Melanoma: A Belvarafenib Expanded Access Program in Patients with RAS/RAF-Mutant Melanoma
Source: Oncologist. 2024 Mar 12;29(6):e811–21. doi: 10.1093/oncolo/oyae018 (PMC11144978; doi:10.1093/oncolo/oyae018)
Supplement: oyae018_suppl_Supplementary_Figures [file oyae018_suppl_supplementary_figures.pdf]

A

**BRAF mutation**<br>PCR + NGS (n=145)<br>NGS only (n=47)		**PCR**			Total
		Positive	Negative	Not examined	
**NGS**	Positive	24	2	20	46
	Negative	0	119	27	146
	Total	24	121	47	192

B

**NRAS mutation**<br>PCR + NGS (n=48)<br>NGS only (n=144)		**PCR**			Total
		Positive	Negative	Not examined	
**NGS**	Positive	5	1	28	34
	Negative	0	42	116	158
	Total	5	43	144	192

Supplementary Figure 1. Comparison of *BRAF* and *NRAS* mutation detection rates using conventional PCR-based assays and Next-Generation Sequencing(NGS)
(A) Concordance of *BRAF* mutation detection rates for conventional PCR-based assay and NGS
(B) Concordance of *NRAS* mutation detection rates between conventional PCR-based assay and NGS

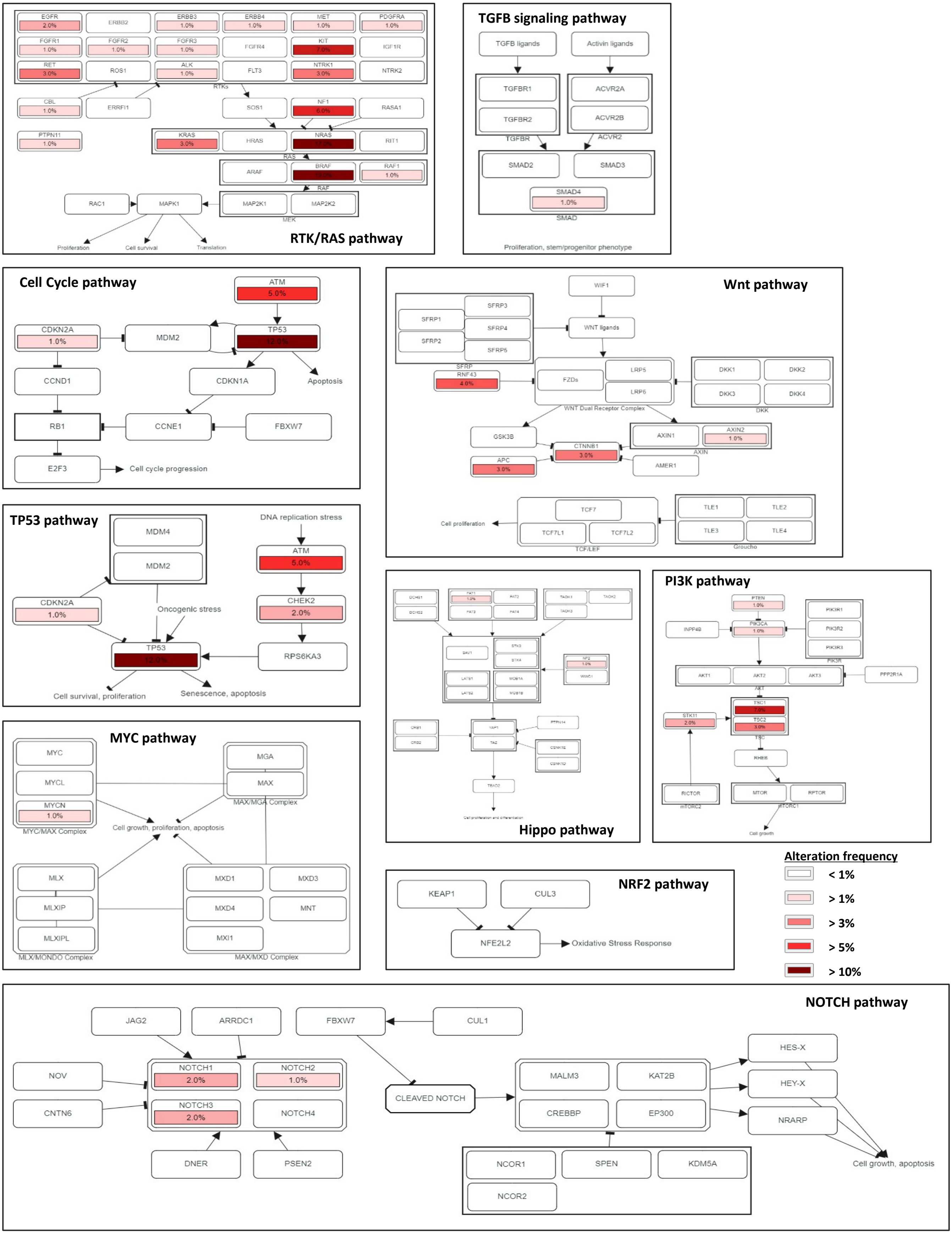

Supplementary Figure 2. Altered signaling pathways in malignant melanoma patients
